# Dynamic expression of Mage-D1 in rat dental germs and potential role in mineralization of ectomesenchymal stem cells

**DOI:** 10.1038/s41598-022-27197-5

**Published:** 2022-12-30

**Authors:** Meng Li, Xia Yu, Yuting Luo, Hongyan Yuan, Yixing Zhang, Xiujie Wen, Zhi zhou

**Affiliations:** 1 Chongqing Key Laboratory of Oral Diseases and Biomedical Sciences, Chongqing Municipal Key Laboratory of Oral Biomedical Engineering of Higher Education, Stomatological Hospital of Chongqing Medical University, Chongqing, China; 2 Department of Orthodontics, Hospital of Stomatology, Southwest Medical University, Luzhou, Sichuan China

**Keywords:** Cell biology, Developmental biology, Molecular biology, Stem cells

## Abstract

Mage-D1 (MAGE family member D1) is involved in a variety of cell biological effects. Recent studies have shown that Mage-D1 is closely related to tooth development, but its specific regulatory mechanism is unclear. The purpose of this study was to investigate the expression pattern of Mage-D1 in rat dental germ development and its differential mineralization ability to ectomesenchymal stem cells (EMSCs), and to explore its potential mechanism. Results showed that the expression of Mage-D1 during rat dental germ development was temporally and spatially specific. Mage-D1 promotes the proliferation ability of EMSCs but inhibits their migration ability. Under induction by mineralized culture medium, Mage-D1 promotes osteogenesis and tooth-forming ability. Furthermore, the expression pattern of Mage-D1 at E19.5 d rat dental germ is similar to p75 neurotrophin receptor (p75NTR), distal-less homeobox 1 (Dlx1) and msh homeobox 1 (Msx1). In addition, Mage-D1 is binding to p75NTR, Dlx1, and Msx1 in vitro. These findings indicate that Mage-D1 is play an important regulatory role in normal mineralization of teeth. p75NTR, Dlx1, and Msx1 seem to be closely related to the underlying mechanism of Mage-D1 action.

## Introduction

Teeth, composed of three mineralized tissues (dentine, cementum, and enamel), constitute important models for gaining insight into the general processes of biological mineralization. Both cell-derived microstructures and extracellular matrix components play critical roles in the preorganization and oriented deposition of calcium phosphate and serve as passive supports in dentine and enamel. However, the mechanism of dental mineralization is still far from being revealed, which restricts the process of dental tissue engineering and tooth regeneration. Ectomesenchymal stem cells (EMSCs) derived from the cranial neural crest are significant in tooth development and dental mineralization. The development of teeth is initiated by epithelial–mesenchymal interactions, which form dental papilla cells and dental sac cells, subsequently forming pulp, dentin, cementum, periodontal ligament and proper alveolar bone, except enamel^1–4^. In our previous studies, EMSCs were obtained from rat embryonic facial process tissue by fluorescence p75 neurotrophin receptor (p75NTR) activated cell sorting, providing a good stem cell model for studies of dental mineralization. Further studies confirmed that p75NTR participates in the regulation of tooth development maybe by changing the activity of the key factor distal-less homeobox/msh homeobox (Dlx/Msx), and melanoma-associated antigen D1 (Mage-D1) seems to be play a role in the differentiation and mineralization of EMSCs^5,6^.

Mage-D1, also known as Dlxin-1 or NRAGE, was first cloned and identified as a new member of the type II melanoma-associated antigen gene family by Pold et al. in 1999^7^. Structurally, Mage-D1 possesses the N-terminal sequence Mage homology domain 2 (MHD2), which is a highly conserved structure of the type II Mage gene family. The C-terminal sequence of 220 amino acids is named the Mage homology domain (MHD) and contains 25 repeats of a WQXPXX sequence in the middle region^7–10^. Studies have confirmed that Mage-D1 can interact with a variety of proteins through its three domains^11^. According to reports, Mage-D1 can not only interact with transcription factors (Dlx/Msx^12,13^) but also interact with nuclear proteins (PCNA^14^, TBX2^15^), cell surface receptors (p75NTR^16^, UNC5H1^17^, ROR2^18^, TrkA^19^) and other proteins involved in cell differentiation, apoptosis, the cell cycle, tumorigenesis and metastasis^20^.

Recently, the effects and mechanism of Mage-D1 in tooth development have been a focus of research. Qi et al. pointed out that Mage-D1 may participate in the proliferation of bone marrow mesenchymal stem cells (BMSCs) and the differentiation of odontoblasts through the NF-κB signalling pathway^21^. Subsequent studies further confirmed that Mage-D1 inhibits the NF-κB signalling pathway by combining with IκB kinase β (IKKβ)^22^, which is a vital regulator of odontoblast differentiation. Liu et al. showed that knocking out Mage-D1 can induce the expression of autophagy-related genes by enhancing the activity and differentiation of osteoclasts, thereby accelerating the process of periodontitis^23^. Our previous studies have shown that Mage-D1 could affect the bone differentiation ability of rat EMSCs by binding to p75NTR^5^. All of these studies suggested that Mage-D1 may play an irreplaceable role in the development of teeth.

In this study, to further explore the specific roles of Mage-D1 in tooth development, we first observed the expression pattern of Mage-D1 in rat dental germ development. Then, we investigated the odontogenesis and mineralization regulation and potential mechanisms of Mage-D1 in vitro through embryonic day 19.5 (E19.5 d) EMSCs.

## Results

### Haematoxylin–eosin (HE) staining during rat dental germ development and immunohistochemistry staining for Mage-D1

**Figure 1:**
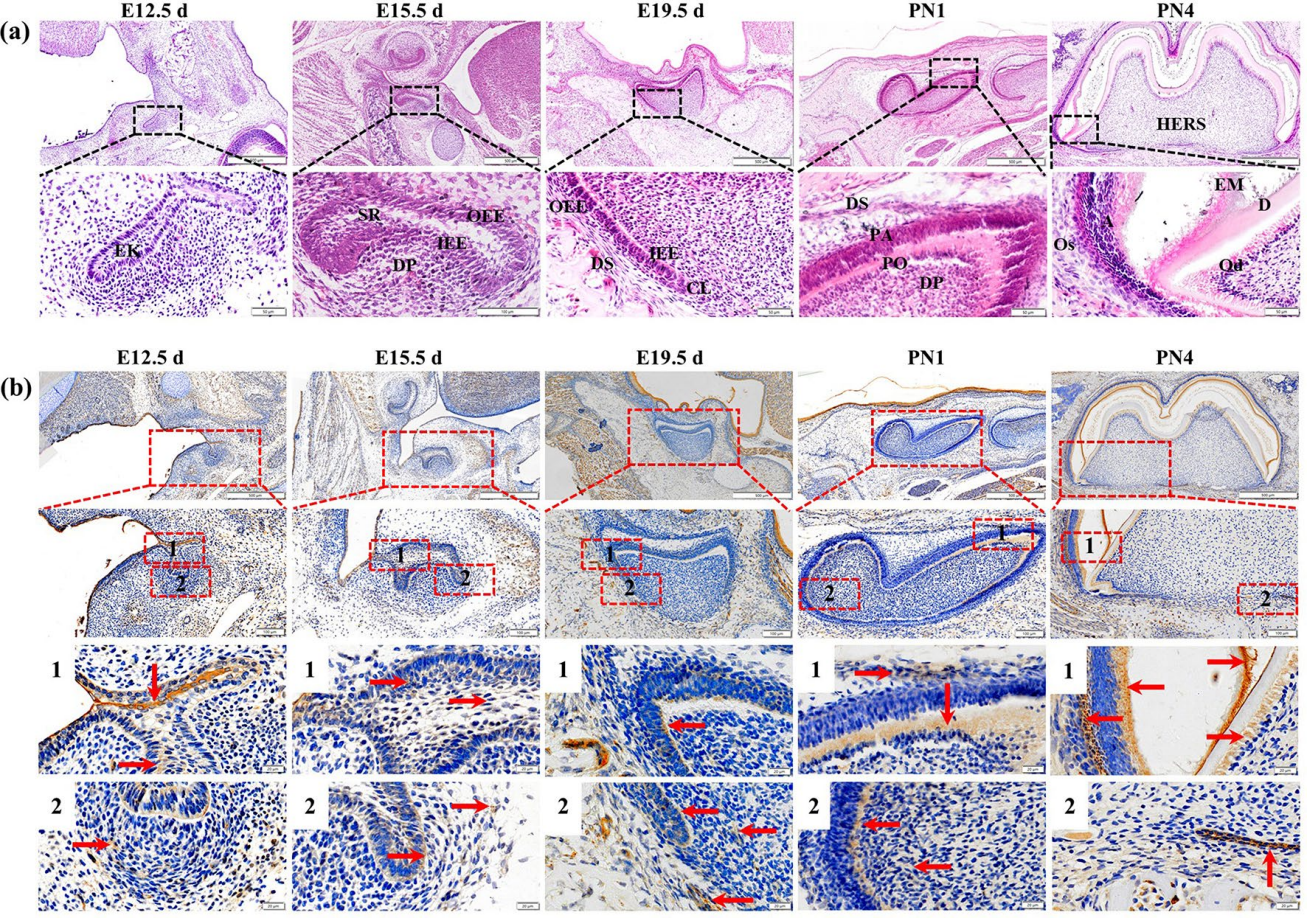
The results of HE staining and expression pattern of Mage-D1 in the stage of rat dental germ development. (**a**) HE staining of first molar in rat at E12.5 d, E15.5 d, E19.5 d, PN1, PN4. (**b**) Mage-D1 expression in first molar of rat at E12.5 d, E15.5 d, E19.5 d, PN1 and PN4 by immunohistochemistry (Red arrow indicates positive area). Scale bars: 50 μm or 500 μm. *EK* enamel organ, *SR* stellate reticulum, *IEE* inner enamel epithelium, *OEE* outer enamel epithelium, *DP* dental papilla, *DS* dental sac, *CL* cervical loop, *PA* preameloblasts, *PO* preodontoblast, *HERS* Hertwig’s epithelial root sheath, *A* ameloblasts, *D* Dentin, *EM* Enamel matrix, *Od* odontoblast, *Os* osteoblast.

HE results (Fig. 1a) showed that the end of the dental plate of the enamel organ swelled into a flower bud shape at E12.5 d, which was the bud stage of dental germ development. Following E15.5 d, it entered the cap phase, and during this period, it was possible to distinguish the components and supporting tissues of the tooth. Then, E19.5 d entered the bell-shaped period. At this time, the enamel organ was divided into four layers: the outer glaze epithelial layer, the inner epithelial layer, the star network layer and the middle layer. Subsequently, the dental germ developed continuously, 1 day after birth (PN1) was in the late bell-shaped stage, and the root of tooth had not yet developed. The morphological development of crown at PN4 was basically complete, inner enamel epithelium and outer enamel epithelium proliferate at the cervical loop, and the epithelial root sheath began to form, which marks the beginning of tooth root development.

Immunohistochemistry (Fig. 1b) was used to further observe the expression pattern of Mage-D1 during dental germ development. During the E12.5 d period, Mage-D1 was strongly expressed in the oral epithelium and outer enamel epithelium instead of the oral mesenchyme, where the pattern is less strong. At E15.5 d, Mage-D1 was widely expressed in the oral epithelium, stellate reticulum, inner enamel epithelium, outer enamel epithelium, and dental sac. However, it was scattered expressed in the dental papilla. During the E19.5 d period, Mage-D1 was strongly expressed in the inner enamel epithelium, cervical loop and dental sac, also scattered expressed dental papilla. At PN1, Mage-D1 was strongly expressed in preodontoblasts, dental sac, and scattered expressed dental papilla. During the PN4 period, Mage-D1 was expressed in ameloblasts, odontoblasts, dental follicle cells, alveolar bone osteoblasts, epithelial root sheaths, and enamel matrix but not in early dentin. In conclusion, the expression position of Mage-D1 on different days of early tooth formation has temporal and spatial specificity.

### Isolation and characterization of rat embryonic EMSCs

**Figure 2:**
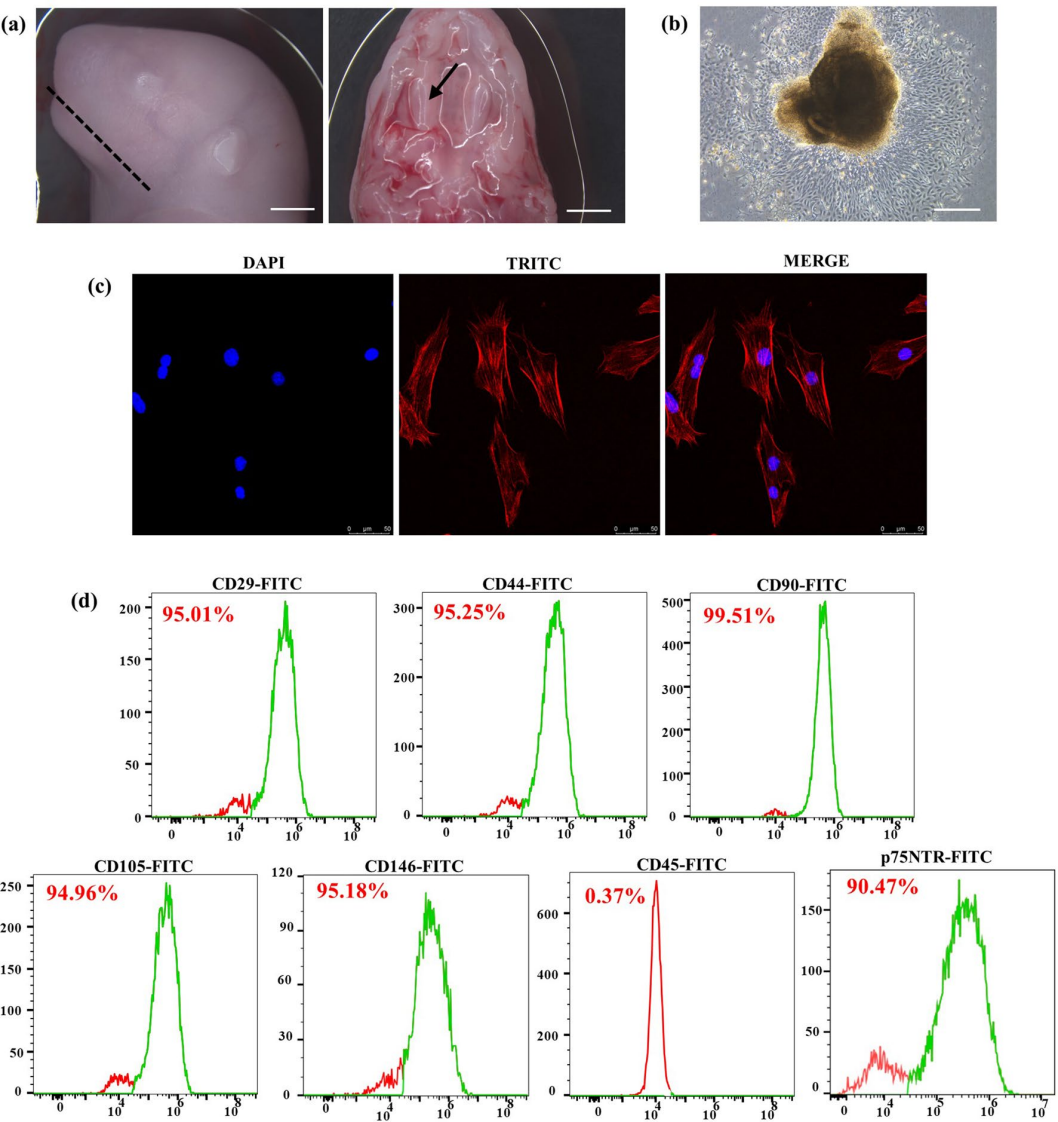
Isolation and characterization of rat embryonic EMSCs. (**a**) The E19.5 d SD rat embryo head and maxillary process. Scale bars: 2.5 mm. (**b**) The E19.5 d EMSCs. Scale bars: 250 μm. (**c**) Phalloidin staining of E19.5 d EMSCs. Scale bars: 50 μm. (**d**) The MSC markers CD29, CD44, CD90, CD105, and CD146 were strongly expressed in EMSCs, while the hematopoietic marker CD45 was hardly detected. The expression of the surface of cranial nerve crest marker p75NTR is 90.47%.

SD foetal rats at 19.5 days of pregnancy were obtained, and the maxilla was separated (Fig. 2a). Then, the maxillary dental germ was obtained, and the EMSCs were successfully isolated and cultured by the tissue block adhesion method (Fig. 2b). The third generation of cells was used for subsequent experiments. The cytoskeleton was stained with phalloidin, which showed that the E19.5 d EMSCs were uniformly long and spindle-shaped and had a fibroblast-like morphology (Fig. 2c). Cell surface antigen was detected by flow cytometry (Fig. 2d). The expression rates of mesenchymal stem cell (MSC) surface markers CD29, CD44, CD90, CD105 and CD146 in E19.5 d EMSCs were 95.25%, 95.01%, 99.51%, 94.96%, and 95.18%, respectively, while the expression rate of the haematopoietic stem cell surface marker CD45 was 0.37%. Moreover, p75NTR, as a marker for isolated cranial neural crest-derived EMSCs^5,24–26^ and the results showed that the expression of p75NTR was 90.47%. These results indicated that the cells used in our experiments were EMSCs.

### Mage-D1 promotes the proliferation and inhibits the migration of EMSCs

**Figure 3:**
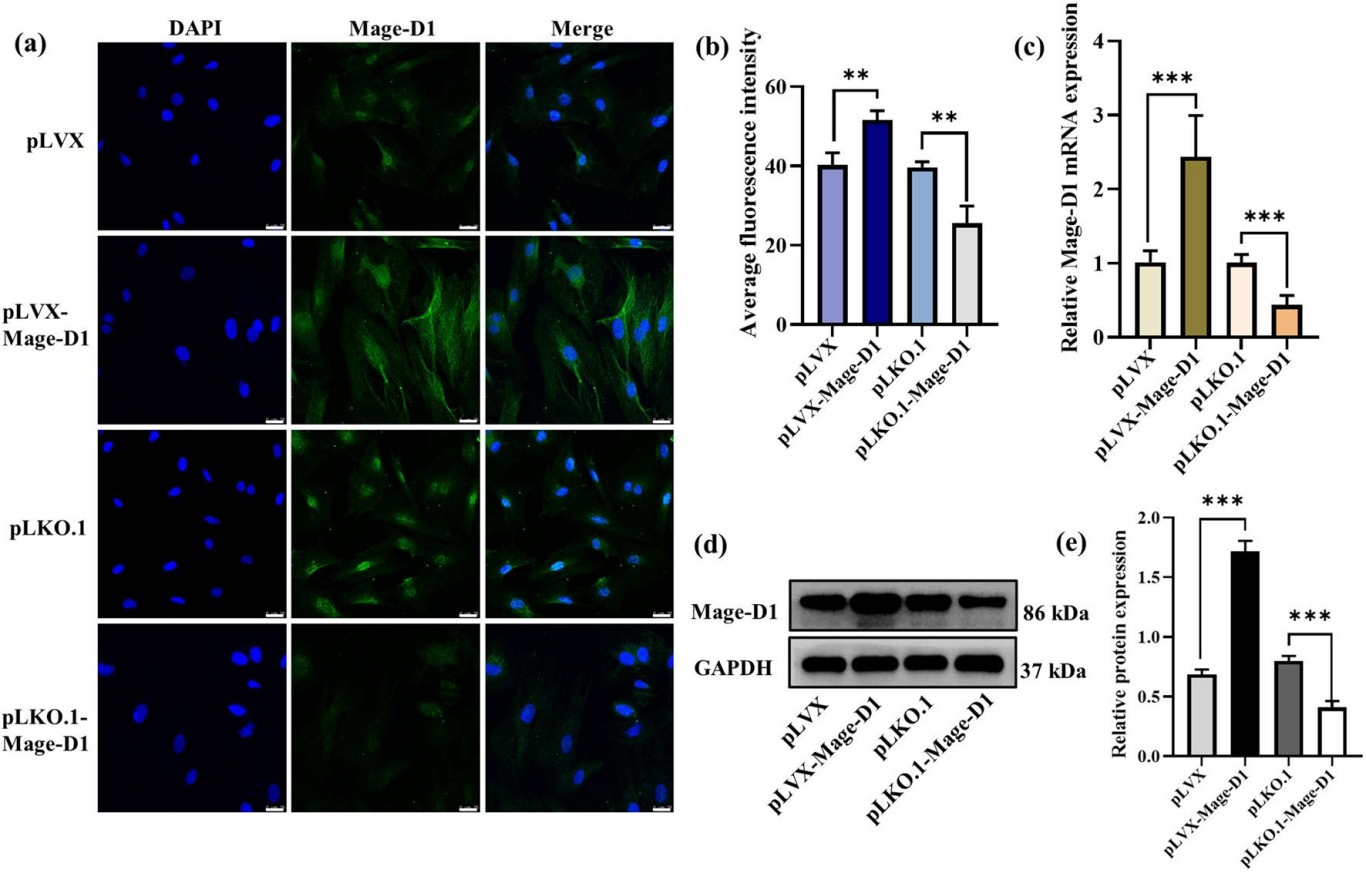
After transfection of E19.5 d EMSCs with Mage-D1 overexpression and silencing plasmids. (**a**) Immunocytofluorescence staining of empty plasmid (pLVX group), transfection with Mage-D1 overexpression plasmid pLVX (pLVX Mage-D1 group), empty plasmid (Plko.1 group) and transfection with Mage-D1 silence plasmid pLKO.1 (pLKO.1-Mage-D1 group); scale bar represents 25 μm. (**b**) Immunofluorescence quantitative statistics of (**a**), statistics are presented in Supplementary Fig. S1 and Supplementary Table 2. (**c**) After transfection of cells with lentivirus, the expression levels of Mage-D1 were examined by real-time PCR normalized to GAPDH. For raw data see Supplementary Table 3. (**d,e**) After transfection of cells with lentivirus, the expression levels of Mage-D1 were detected by Western blot analysis, GAPDH used as the reference gene. Full-length blots are presented in Supplementary Fig. S2. (**P < 0.01, ***P < 0.001).

**Figure 4:**
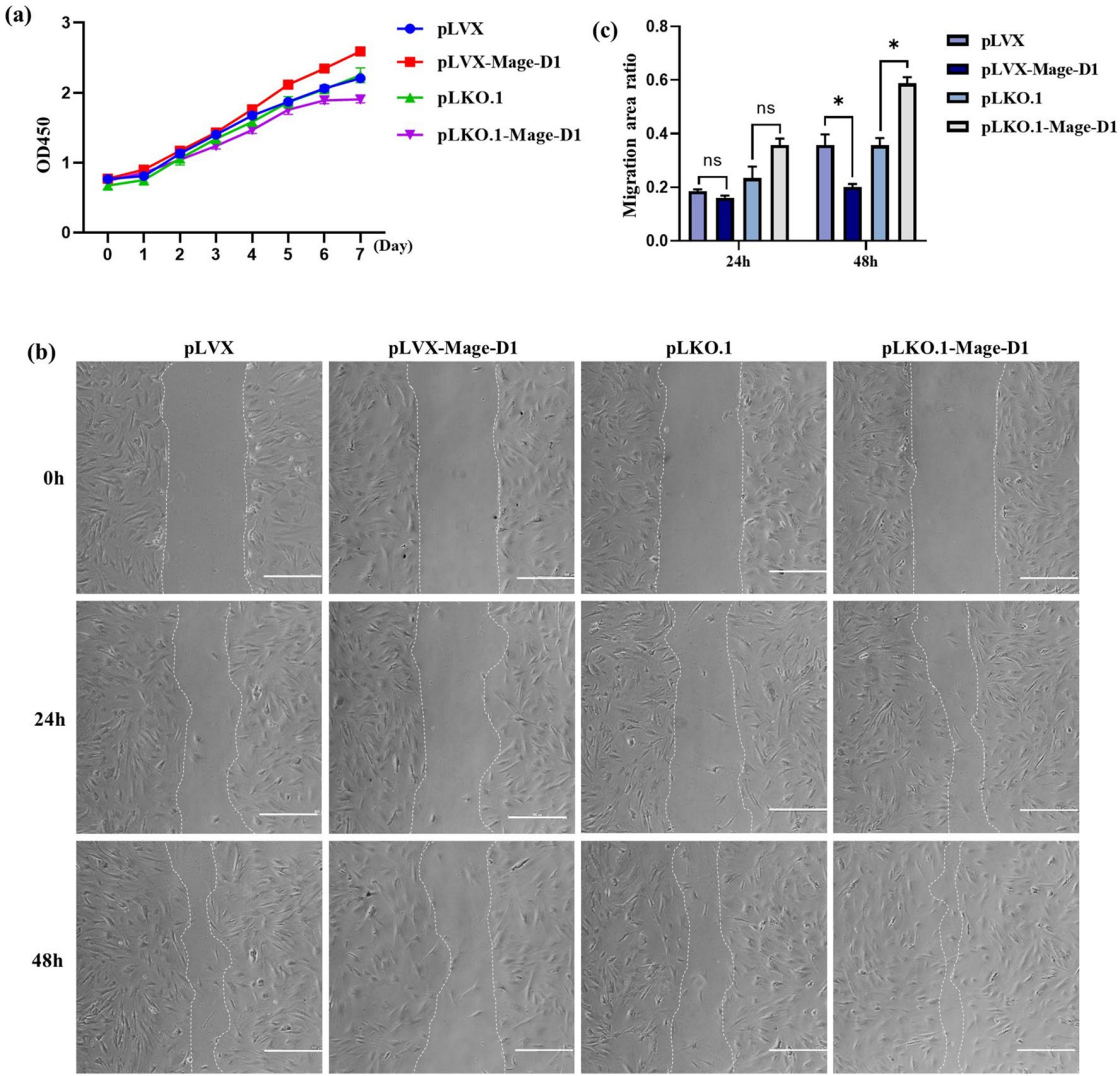
Regulation of Mage-D1 influence the proliferation capacities and migration capacities of E19.5 d EMSCs. (**a**) The proliferation rate was assessed by CCK-8 cultured for 8 days. For raw data see Supplementary Table 4. (**b**) The migration rate was assessed for two consecutive days. Scale bar represents 500 μm. (**c**) The migration rate quantitative statistics of (b). For raw data see Supplementary Fig. S3 and Supplementary Table 5. (ns represents no significance, *P < 0.05).

We successfully constructed Mage-D1 overexpression and silenced lentivirus-transfected EMSCs in vitro. Immunofluorescence results (Fig. 3a,b) showed that the fluorescence staining intensity of Mage-D1 in the overexpression group was significantly higher than that in the control group especially in the cytoplasm. Polymerase chain reaction (PCR) and western blot (WB) results (Fig. 3c–e) showed that the expression of Mage-D1 protein and gene in the overexpression group was significantly higher than that in the control group, and the silencing group was the opposite. The cell counting kit-8 (CCK-8) assay results (Fig. 4a) showed that Mage-D1 positively regulated the proliferation of EMSCs, but further scratch assay results (Fig. 4b,c) showed that Mage-D1 negatively regulated the migration of EMSCs.

### Mage-D1 positively regulates the osteogenesis of EMSCs

**Figure 5:**
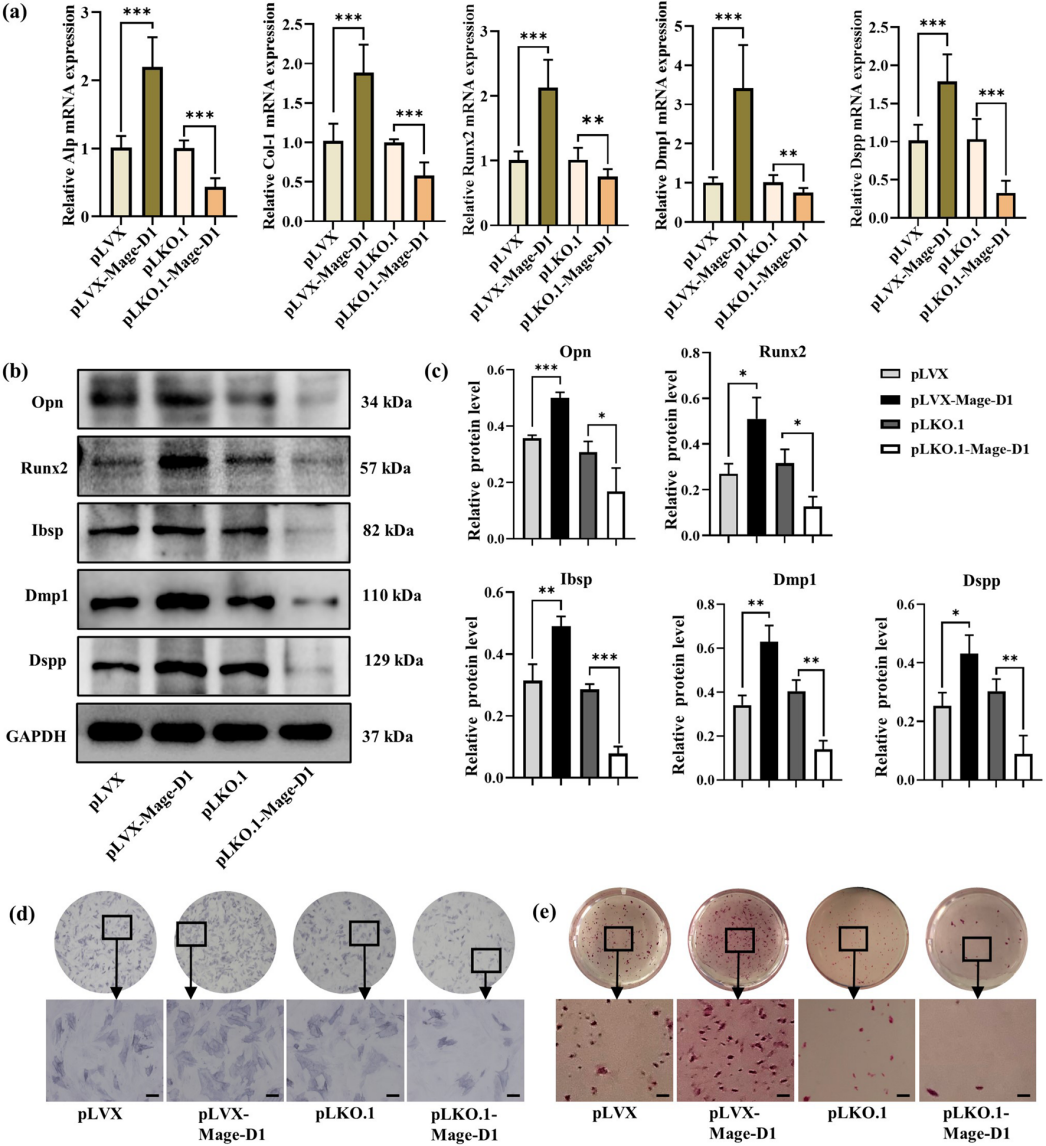
Overexpression and silencing of Mage-D1 affects the mineralization regulation of E19.5 d EMSCs. (**a**) Under induction with mineralized culture medium for 7 d, the expression levels of Alp, Runx2, Col-1, Dmp1, Dspp were examined by real-time PCR normalized to GAPDH. For raw data see Supplementary Table 3. (**b,c**) Under induction with mineralized culture medium for 14 d, the expression levels of Opn, Runx2, Ibsp, Dmp1, Dspp were detected by Western blot analysis, GAPDH used as the reference gene. Full-length blots are presented in Supplementary Fig. S4. (**d**) Under induction with mineralized culture medium for 14 d, Alp staining was used to detect their potential of differential mineralization. Scale bar represents 50 μm. (**e**) Under induction with mineralized culture medium for 21 d, alizarin red staining was used to detect their mineralized nodules. Scale bar represents 200 μm. (**P < 0.01, ***P < 0.001).

According to reports, Mage-D1 is involved in the development and mineralization of teeth^5^. Therefore, we explored whether Mage-D1 is related to the osteogenesis and odontogenesis of EMSCs. After overexpressing Mage-D1 and culturing with mineralization induction medium, the mRNA levels (Fig. 5a) of mineralization-related factors alkaline phosphatase (Alp), runt-related transcription factor 2 (Runx2), collagen type-1 (Col-1) and odontoblast-like differentiation markers dentin sialoph-osphoprotein (Dspp) and dentin matrix protein 1 (Dmp1) in EMSCs were increased. The protein levels (Fig. 5b,c) of Runx2, bonesialoprotein II (Ibsp), osteopontin (Opn), Dspp and Dmp1 were also increased. Further results for both Alp staining (Fig. 5d) to detect mineralization ability and alizarin red staining (Fig. 5e) to detect mineralized knots showed that Mage-D1 enhanced mineralization. At the same time, after we silenced Mage-D1 and cultured it with mineralization induction medium, the results showed that Mage-D1 inhibited the mineralization and osteogenic ability of EMSCs. Briefly, Mage-D1 is closely related to the development of teeth and positively regulates the osteogenesis and tooth-forming ability of EMSCs.

### Potential mechanism of Mage-D1 in regulating mineralization

**Figure 6:**
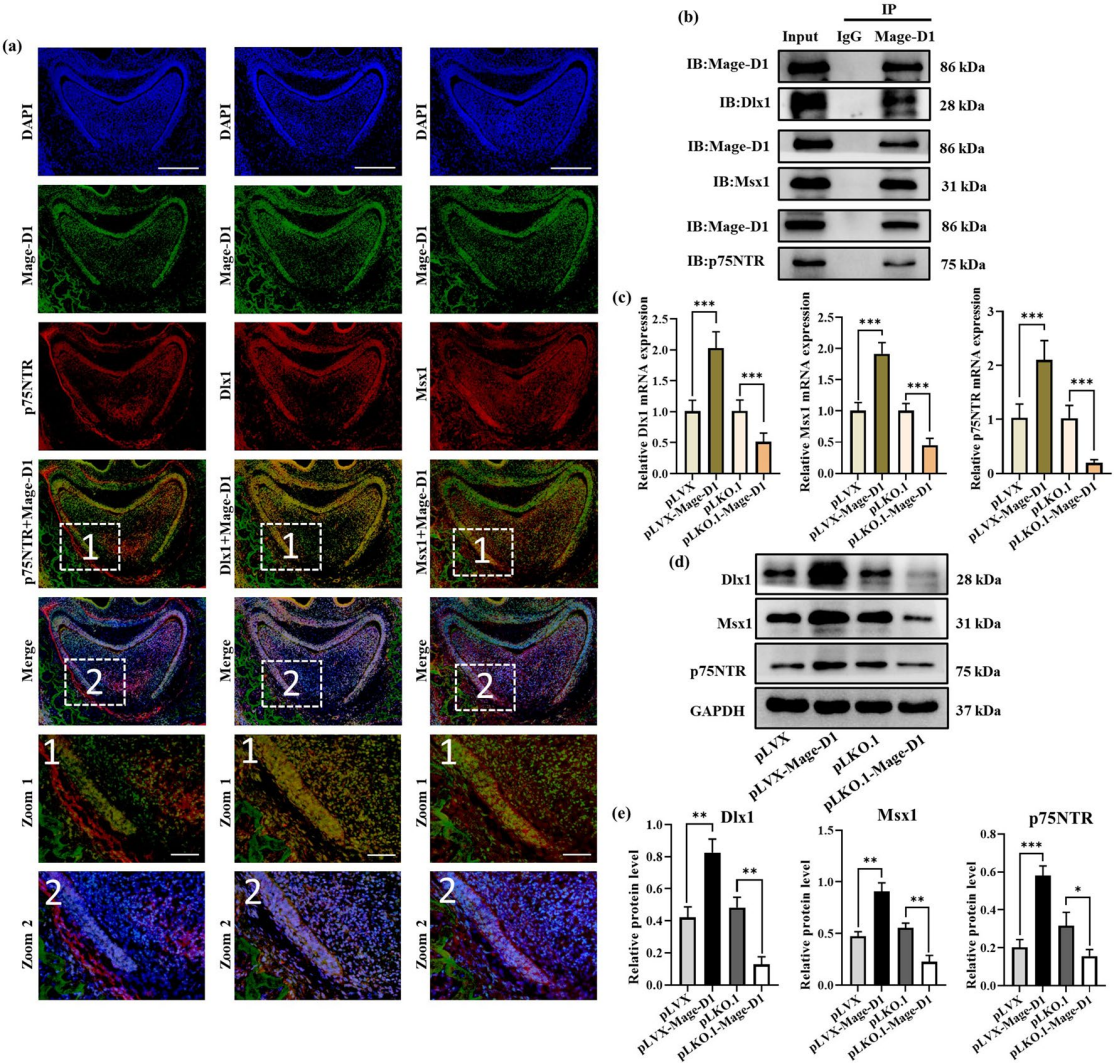
Investigations revealing the potential mechanism of Mage-D1 in mineralization. (**a**) Immunofluorescence double staining of Mage-D1, p75NTR, Dlx1 and Msx1 at E19.5 d rat dental germ. Scale bar represents 200 μm or 50 μm. (**b**) Co-immunoprecipitation shows that Mage-D1 can bind to p75NTR, Msx1, Dlx1 based on E19.5 d EMSCs. Full-length blots are presented in Supplementary Fig. S5. (**c**) Under induction with mineralized culture medium for 7 d, the expression levels of p75NTR, Msx1, Dlx1 were examined by real-time PCR normalized to GAPDH. For raw data see Supplementary Table 3. (**P < 0.01, ***P < 0.001). (**d,e**) Under induction with mineralized culture medium for 14 d, the expression levels of p75NTR, Msx1, Dlx1 were detected by Western blot analysis, GAPDH used as the reference gene. Full-length blots are presented in Supplementary Fig. S6.

Mage-D1 is involved in the tooth formation and osteogenesis process of EMSCs, but its specific mechanism is still unclear. Zhao et al. speculated that Mage-D1 plays a bridge role between the cell membrane receptor p75NTR and the nuclear transcription factor Dlx/Msx^6^. At E19.5 d, the key time points of rat dental germ development, our immunofluorescence double staining results showed that p75NTR and Mage-D1 were well co-localized in the inner enamel epithelium, outer enamel epithelium, and the apical of dental papilla. The expression of p75NTR in the dental sac was stronger than that of Mage-D1. The expression location of Dlx1 and Mage-D1 is very similar. They are well co-localized in the inner enamel epithelium, outer enamel epithelium, dental papilla and dental sac. Msx1 and Mage-D1 are well co-localized in dental papilla and cervical loop. Then the immunoprecipitation results (Fig. 6b) showed that Mage-D1 not only binds to p75NTR but also to Dlx1 and Msx1. Further studies have shown that after overexpression of Mage-D1, the gene and protein levels of p75NTR, Dlx1, and Msx1 were increased, after silencing Mage-D1, the gene and protein contents of p75NTR, Dlx1, and Msx1 were decreased (Fig. 6c–e). These results suggested that the involvement of Mage-D1 in the process of osteogenesis or tooth formation may be closely related to p75NTR, Dlx1, and Msx1.

## Discussion

Mage-D1 plays an essential role in life activities, including the cell cycle, cell adhesion, cell differentiation, apoptosis, and some tumour events, such as tumour occurrence, invasion, and metastasis^11^. Our research revealed that Mage-D1 is expressed throughout the process of dental germ development and that Mage-D1 plays a key role in the proliferation, migration and differential mineralization of EMSCs. The potential mechanism of Mage-D1 involvement in differential mineralization seems to be related to p75NTR, Dlx1 and Msx1.

Mage-D1 expression is regulated by strong posttranscriptional regulation during murine embryogenesis and presents spatiotemporal tissue specificity^27^. However, the expression of Mage-D1 in dental germs has not yet been clearly elucidated, so our research investigated it first. As the results showed, Mage-D1 was strongly expressed in the oral epithelium, outer enamel epithelium instead of the oral mesenchyme at E12.5 d, which suggested that Mage-D1 may be connected with the initiation of tooth development. During bell stage, the cells in different parts have specific phenotypes and have the ability to form corresponding tooth tissues. The early bell stage is the stage of odontoblast formation and dentin matrix deposition during tooth development^28^. The outer layer cells of dental papilla differentiated into high columnar odontoblasts to form mineralized dentin in the future. Mage-D1 was strongly expressed in the dental sac and cervical loop at E19.5 d, which suggested that Mage-D1 may be involved in the development of cement, periodontal ligament, alveolar bone and the formation of epithelial root sheath in the future. It also can be seen that Mage-D1 was also obviously expressed in the inner enamel epithelium at E19.5 d. With the development of enamel organ, the inner enamel epithelium differentiates into ameloblasts and participates in the mineralization of teeth. Then the expression of Mage-D1 strongly expressed in preodontoblast, and dental sac at PN1, which suggested that Mage-D1 may be associated with the formation of dentin. Mage-D1 is widely expressed in ameloblasts, odontoblast, alveolar bone osteoblasts, enamel matrix and epithelial root sheath at PN4, which suggests that Mage-D1 may also be involved in the development of enamel, dentin and roots. The process of tooth development is a process of enamel dentin alternate mineralization. In short, the above studies showed that Mage-D1 was expressed with temporal and spatial specificity in dental germ development and related to the development of tooth mineralization.

EMSCs provide a profitable in vitro stem cell model for the study of dental morphogenesis^12^. The enamel organ entered a mature stage at E19.5, the late bell-shaped stage. Histological results showed that the expression of Mage-D1 in dental germ was at a high level at E19.5 d, which was more suitable for studying the regulatory function of Mage-D1 in vitro. Previous studies have shown, p75NTR, as a marker for isolated cranial neural crest-derived EMSCs^5,24–26^, was expressed at a high rate and with higher purity in E19.5 d EMSCs^5^. In this study, EMSCs were extracted from foetal rats at E19.5 d SD.

The migration and proliferation of cells are important for epithelial-mesenchymal transfer and metabolism during tooth development and involve many gene-regulating processes^29,30^. Our studies showed that Mage-D1 can inhibit the migration of EMSCs, which is consistent with the migration inhibition result of Mage-D1 in glioma stem cells^26^, human breast cancer cells^31^, HeLa cells^32^, melanoma or pancreatic cancer cells^33^ and many other tumour cells. Cell migration is a critical physiological event associated with four other main cellular processes: adhesion, proliferation, differentiation, and death. Wu et al. reported that Mage-D1 can suppress the differentiation of mouse dental papilla cell-23(MDPC-23) cells and odontoblast-lineage cell (OLCs) by interrupting the premature formation of dentin^22^. The migration inhibition effect of Mage-D1 in EMSCs may affect the stages of dentin differentiation or other processes, followed by affecting the development of teeth. Moreover, Mage-D1 can promote the proliferation of EMSCs, which is similar to the effect of Mage-D1 on the proliferation of dental pulp cells revealed by Qi et al.^21^. As the study of Gina at el. showed that siRNA knockdown of Mage-D1 increased the proliferation of primary calvarial osteoblasts^34^. Jiang et al. also indicated that interference with Mage-D1 expression could reduce the proliferation of human gastric cancer cell lines^35^. In oesophageal carcinomas, Mage-D1 accelerates cell proliferation by stabilizing proliferating cell nuclear antigen (PCNA) in a ubiquitin–proteasome pathway^36^. Mage-D1 can interact with a variety of proteins with different functions^14^. For example, the interaction of p75NTR can regulate apoptosis^37^, the interaction of p53 can regulate the cell cycle^38^. It is not surprising that the roles of Mage-D1 vary because of cell type specificity^39^. We also found that a large number of subjects showed the inhibitory proliferative effect of Mage-D1; for instance, Mage-D1 inhibited the proliferation of breast cancer cells when ectopically expressed^40^. Some studies also indicated that the protein complex with Mage-D1, Dlx5 and Necdin could arrest the proliferation of osteogenic cells^41^. In short, Mage-D1 plays complex and diverse roles in life activities, the impact of Mage-D1 on the proliferation and migration of EMSCs is closely related to tooth development.

Mage-D1 is expressed in adherent cells rather than haematopoietic cells in bone, including osteoblastic and chondrogenic cells^42^. Our research also found that Mage-D1 is strongly expressed in various mineralization-related cells during the development of dental embryos. We further investigated the regulatory role of Mage-D1 in mineralization and tooth formation. Dspp belongs to dentin-specific proteins^43^. Dmp1, Ibsp, and Opn are mineralized tissue-specific proteins, and they play important roles in inducing cell differentiation and promoting dentin mineralization. Col-1 and Runx2 proteins are predominantly expressed during the earlier phases of proliferation and maturation, and Alp and Opn are expressed after the middle phase^4^. All of them play considerable roles in differential mineralization during the developmental stages of teeth and maxillofacial structures^5^. The protein and gene expression levels of relevant mineralization and odontogenic factors were increased in EMSCs after overexpression of Mage-D1, and the above results were reversed after silencing Mage-D1, which suggested that Mage-D1 positively regulated osteogenic differentiation in rat EMSCs. Gina at el. observed that siRNA knockdown of Mage-D1 increased the early expression of Alp, but decreased mineralized nodule formation, most importantly, that remarkable descent bone mineral density (BMD) in Mage-D1-deficient mice^34^. In addition, as the study of Liu et al. showed that the lack of Mage-D1 resulted on an enhanced osteoblast differentiation via ALPL and BGLAP, and also in a higher osteoclast differentiation via Ctsk and ACP5^44^, and the latter exceeding the former, the bone degradation rate was bigger than mineralization rate, resulting in bone lose. In human osteosarcoma cells (MG63 cells), the expression of Runx2 was concurrently increased with Dlx5, Mage-D1 and Necdin^41^. Both bone tissue and teeth are mineralized tissues, so there are many commonalities in their biological characteristics and matrix formation. However, there are also published articles that showed just the opposite, as Qi et al. where in mice DPCs Mage-D1 expression reduced osteogenic differentiation via NF-κB signaling^21^. The discrepancy could be caused by the method to knock down/knock out Mage-D1 expression. In our study, endogenous expression of Mage-D1 was stably downregulated by lentivirus-mediated shRNA. In the study of Gina at el., siRNA transient transfection was used to knock down the expression level of Mage-D1 in the undifferentiated osteoblasts^34^. In the reaserch of Liu et al. the osteoblasts were isolated from Mage-D1 knockout mice. In addition, the diverse effects of Mage-D1 could be caused by varies with the cell types, such as osteoblasts, EMSCs, DPCs. Moreover, the time point of EMSCs were extracted could be affected the obtained results^1^, which suggesting the complex and diverse roles of Mage-D1 in osteogenesis and odontogenesis. Furtherlly, Mage-D1 can change its cellular localization under the stimulation of some factors; for example, the interaction of nerve growth factor (NGF) with p75NTR was responsible for the translocation of Mage-D1 from the cytoplasm to the cell membrane^45^, which represents the diverse functions of Mage-D1.

The expression of Mage-D1 in the mature rat brain was similar to that of p75NTR, but Mage-D1 was more widely distributed, suggesting that Mage-D1 may also participate in other signalling pathways^46^. Moreover, in mouse dental germ, Mage-D1 was intensity expressed in the dental sac and dental papilla, analogous to that of p75NTR, which implied that Mage-D1 might be associated with P75NTR during tooth development^6^. The Dlx/Msx family has been shown to play a role in tooth development and regulate mineralization-related genes^47–49^. Msx1 participates in initial dental embryogenesis and regulates the proliferation and differentiation of mouse dental mesenchymal cells^50^. The presence of Dlx1 is a key factor for normal tooth development, especially for the development of maxillary molars^51^. It is amazing that most Dlx/Msx family proteins are connected with Mage-D1. Mage-D1 may be a common transcriptional regulator mediated by Dlx/Msx homologous domain proteins^42,52^. Previous studies speculated that Mage-D1 acts as a bridge between p75NTR and Dlx/Msx^5,6^. However, their research did not point out the relationship between them in detail, further studies are needed to determine the exact mechanism underlying this process. Our immunofluorescence double staining results showed that the expression location of Mage-D1 and p75NTR, Dlx1, Msx1 in rat dental germ is greatly similar, suggesting that they may exist a certain correlation. These are consistent with the research reported by Zhao et al.^6^ Therefore, we further observed the binding in vitro by coimmunoprecipitation, which verified that Mage-D1 could bind to p75NTR, Dlx1 and Msx1 with EMSCs as the cell model. Ultimately, the protein and gene levels of p75NTR, Dlx1, and Msx1 were significantly increased after overexpression of Mage-D1, and the opposite results were obtained after silencing. p75NTR is a membrane receptor protein, and Dlx1 and Msx1 are intranuclear transcription factors. Mage-D1, a significant protein that can change subcellular localization and expression levels in response to external signal stimulation, may transmit signals from outside the cell to transcriptional proteins in the plasmatic nucleus by binding to the three proteins; therefore, a series of cellular activities are triggered. The involvement of Mage-D1 in the regulation of tooth development closely related to the binding and signalling of Mage-D1, which affected by both Mage-D1 protein expression and intracellular localization, but the specific signalling mechanism needs to be explored further. Our study further shows that the relationship between Mage-D1 and p75NTR is not only a simple upstream–downstream relationship, but more like a synergistic and mutually restraining relationship. Mage-D1 and Dlx1/Msx1 maybe shows upstream–downstream relationship. It is attributed to the diverse biological functions of Mage-D1 and complex signal regulation mechanism, which can be seen that the mechanism by which Mage-D1 is involved in mineralization of EMSCs is diverse and complex.

In addition, in our study, we can see that oral epithelium have a strongly Mage-D1 expression pattern in every period. But these cells doesn´t differentiate to ameloblasts like the inner enamel epithelial cells. Further investigation is needed to clarify the direct effect of Mage-D1 on mineralization, probably some kind of signaling pathway communication between epithelial and mesenchy mal cells is needed for ameloblast and odontoblast differentiation. Previous studies revealed that p75NTR targets the Wnt/β-catenin pathway and positively regulates EMSCs osteogenic diferentiation, suggesting that Wnt/β-catenin pathway may be involved in the development of teeth^53^. The data of Zhou et al. indicated that Mage-D1 extremely may be a pivotal factor involved in Wnt/β-catenin signal pathway, mediating nuclear translocation of β-catenin^54^. Therefore, we will continue to study the direct pathway of Mage-D1 involved in tooth mineralization.

In summary, our studies pointed out that Mage-D1 was differentially expressed in time and space during tooth development and negatively regulated the migration of EMSCs but positively regulated the proliferation and mineralization of EMSCs. Further studies suggested that Mage-D1 may be closely related to p75NTR, Dlx1 and Msx1 in tooth development. Taken together, our results show that Mage-D1 is play a regulatory role in normal mineralization of teeth. This novel finding may prompt future studies devoted to exploring how Mage-D1 functions and use of its molecular mechanism in dental histological engineering.

## Materials and methods

Firstly, we confirm that all methods were performed in accordance with the relevant guidelines.

### Experimental animals

The existence of a vaginal plug of Sprague–Dawley (SD) rats was treated as E 0.5 d. One day after birth was recorded as PN1. All experiments were conducted in accordance with the scheme approved by the Ethics Committee of Southwest Medical University (The number accepted is 201,903–187, the original document is shown in related files), including any relevant details, and we confirmed that all experiments were performed in accordance with relevant guidelines and regulations.

### HE and immunohistochemistry staining

The SD rat embryo heads or maxillas of E12.5 d, E15.5 d, E19.5 d, PN1, and PN4 (n = 3 each group) were collected and fixed with 4% paraformaldehyde (Solarbio, Beijing, China) for 24 h. The maxilla samples were demineralized with ethylenediaminetetraacetic acid (EDTA) decalcifying solution (Solarbio). Then, the samples were dehydrated by alcohol, embedded in paraffin and sliced into 5 μm tissue sections. HE staining was carried out according to the manufacturer’s protocols of HE Staining Kit (Solarbio). After dewaxing and rehydration by xylene and gradient alcohol, the slides were dyed with hematoxylin solution for 3 min, then rinsed with water for 3 min to remove float color. Then the slides were dyed with eosin solution for 30 s and rinsed with water for 3 s. Finally, dehydrated with gradient alcohol, transparented with xylene and sealed treatment. Scanning and analysis of staining results were performed with a slice digitizing scanner (OLYMPUS, Japan). For immunostaining, briefly, after dewaxing and rehydration, the slides were repaired with sodium citrate antigen repair solution (Bioss, Beijing, China), and then incubated with 3% H_2_O_2_ solution to eliminate endogenous peroxidase activity. The slides were blocked with goat serum (Bioss) and then incubated with the rabbit polyclonal antibody to Mage-D1 (1:100, Biorbyt, Cambridgeshire, UK) at 4 °C overnight. Next day, the slides were incubated with an anti-rabbit secondary antibody (1:3000, Bioss), followed by colouring with diaminobenzidine (DAB) (Zsbio, Beijing, China). Finally, cell nuclei were counterstained with haematoxylin (Solarbio). Following dehydrated, sealed and other steps are the same as HE staining.

### Isolation and culture of E19.5 d EMSCs

The isolation and culture of E19.5 d EMSCs were carried out as previously described^1,5^. The embryonic maxillofacial process of E19.5 d SD embryo rats was dissected, and the maxillary dental germ was taken. The minced tissue was placed directly in a sterile petridish using the tissue block adherence method. Then, the complete culture medium (composed of 89% Dulbecco’s modified eagle medium/F12 (Sigma, Darmstadt, Germany), 10% foetal bovine serum (Ausgenex, Gold Coast, Australia) and 1% penicillin–streptomycin liquid (Solarbio)) was gently added and cultured in a humidified incubator at 37 °C and 5% CO_2_ for approximately 3 days. After the cells had fully crawled out, routine follow-up cell passaging treatments were carried out.

### Phalloidin staining

After the cells reached 70% confluence in a six-well plate, they were fixed with 4% paraformaldehyde for 30 min and washed gently with PBS buffer three times, then 0.1% Triton X-100 (Solarbio) was added to break the membrane for 20 min and washed again. Approximately 100 μm of phalloidin working solution (Sigma) was added, followed by incubation for 2 h at room temperature. The cell nucleus was stained with 4′,6-diamidino-2-phenylindole dihydrochloride solution (DAPI) (Solarbio) and incubated for 10 min in the dark. Finally, the cytoskeleton was observed with a confocal laser scanning microscope (CLSM) (Leica CS SP8, Heidelberg, Germany).

### Flow cytometry identification

Approximately 5 × 10^5^ cells were collected in each group, and then we detected cell surface markers by flow cytometry. The cells were fixed with 4% polyoxymethylene for 15 min, and then primary antibodies (mouse monoclonal antibody to CD44, CD29, CD90, CD105, CD146 and p75NTR) (1:100; Santa Cruz Biotechnology, Texas, USA) were added and incubated overnight at 4 °C. The anti-mouse secondary antibody/FITC (1:100, Bioss) was added the next day and incubated for at least one hour. The cells were then analysed by CytoFLEX flow cytometry (Beckman Coulter, California, USA).

### Transfection of Mage-D1-overexpressing and Mage-D1-silenced plasmids

The full-length coding region of rat Mage-D1 was amplified by PCR and cloned into the vector pLVX-puro for expression. The specific primers were as follows: 5′- ACACTCGAGATGGCTCAGAAACCGGACGGCG-3′ (forward) and 5′-CTGAAT TCTTACTCAACCCAGAAGAAGCCAATGGCACCG-3′ (reverse). The plasmids were cotransfected with psPAX2 and pCMV-VSV-G packaging plasmids into HEK-293 T cells with active growth by Lipofectamine 2000 (Invitrogen, Massachusetts, USA). The virus-containing supernatant was collected at 2 to 3 days after transfection, which was used to infect the target cells, the E19.5 EMSCs. Then the six-well plate cells reached 70% confluence, appropriate amount of virus solution and 8 μg/ml polybutene (Solarbio) was added to the cell medium. After 24 h, replace the culture medium containing virus solution with complete culture medium. Then the target cells were continuously screened for about 7 days with 4 μg/ml puromycin (Solarbio). The stable Mage-D1 overexpression of EMSCs was established for further research. To knock down the expression of endogenous Mage-D1, plasmids were established with pLKO.1. The sequences were as follows: 5′-AAGGTGGCCTTTAAGTCACAG-3′. The packaging of these knockdown lentiviruses is similar to that of overexpression lentiviruses.

### Immunofluorescence staining

The transfected cells were plated on a cell slide until the cells reached 70% confluence and then fixed with 4% paraformaldehyde, and then 0.1% Triton X-100 (Solarbio) was added to break the membrane for 20 min, followed by appropriate amount of 5% goat serum (Bioss) was added to each slide to sealed for 1 h at room temperature. After removing the sealing liquid, incubation with the rabbit polyclonal antibody to Mage-D1 (1:100, Biorbyt) overnight at 4 °C. The anti-rabbit secondary antibody/FITC (1:100, Bioss) was added the next day and incubated at room temperature for 30 min. For immunofluorescence double staining of paraffin sections, the steps of dewaxing to blocked were the same as those of immunohistochemistry. Then, two heterologous primary antibodies were diluted and mixed with PBS (the rabbit polyclonal antibody of Mage-D1,1:100, Biorbyt; the mouse monoclone antibody of 75NTR, 1:100, Santa Cruz; Dlx1, 1:100, Santa Cruz; Msx, 1:100, Santa Cruz), followed added to the tissue surface and incubated at 4 °C overnight. Next day, both the anti-rabbit secondary antibody/FITC (1:100, Bioss) of anti-mouse/Cy3 (1:100, Bioss) was added and incubated at room temperature for 1 h. The cell nuclei were counterstained with DAPI. Finlly sealed the cell slide with antifluorescent quencher and observed under CLSM (Leica).

### CCK-8 proliferation and scratch test

Briefly, the transfected EMSCs were seeded in a 96-well plate at 2 × 10^3^ cells/well (Corning). Starting on the second day, we detected cell proliferation by using the CCK-8 assay for 8 consecutive days according to the manufacturer’s instructions. The number of viable cells in each well was determined by measuring the absorbance at 450 nm wavelength with a microplate reader (Varioskan LUX Multifunctional Enzyme Marker, Thermo Fisher Scientific, California, USA). Cell proliferation was expressed as the mean ± standard deviation of the absorbance of 5 wells in each group. For scratch test, the transfected cells were spread in a six-well plate until full. Then, we changed to serum-free medium and used a 200 μL pipette tip to mark the inside of the well plate at least three traces and cultured the cells in a humidified incubator at 37 °C and 5% CO_2_. Photos were taken at 0 h, 24 h, and 48 h by inverted phase contrast microscope (Zeiss, Jena City, Germany). ImageJ software (1.8.0 for Microsoft, National Institutes of Health, Bethesda, USA) was used to analyse cell migration area ratio in each group.

### Real-time PCR assay

General RNA of every group of cells was obtained by Trizol reagent (Invitrogen)^55^. After the cells were lysed by Trizol reagent, the supernatant containing RNA was separated by chloroform, and then isopropyl alcohol was added to precipitate RNA, which was washed with 70% ethanol, and finally RNA was dissolved in non-enzymatic water. RNA was reverse-transcribed into cDNA using the PrimeScript™RT reagent Kit with gDNA Eraser (TaKaRa, Kusatsu, Japan) according to the manufacturer’s instructions. The quantities of RNA and cDNA were detected with the molecular devices (Nanodrop 2000, Thermo Fisher Scientific). Quantitative real-time PCR was carried out by Fluorescence quantitative PCR instrument (Bio-Rad, California, USA) using SYBRII qPCR master mix reagent (TaKaRa) and running with a 20 µl reaction system. There were at least 3 secondary wells per group. At the same time, the GAPDH gene was used as a control. Primer information for related genes is shown in Supplementary Table 1.

### Co-immunoprecipitation (Co-IP) and WB

Total cell proteins were retrieved with cell lysis buffer (Beyotime), and the protein level was measured by the BCA kit (Bioworld, Beijing, China). For coimmunoprecipitation, first, the magnetic beads (Bio-rad) were incubated with rabbit polyclonal antibody to Mage-D1 (1:200, Proteintech, Chicago, USA) for 2 h, followed by the addition of protein lysate and incubation overnight at 4 °C. The unreacted proteins were separated magnetically and discarded the next day. Western blot was conducted as described previously. The protein samples were separated by SDS–PAGE and transferred to PVDF membranes (Pierce, Dallas, USA). The blots on the membranes were cut prior to hybridization with antibodies during blotting. Primary antibodies against Mage-D1 (1:1000; Biorbyt), p75NTR (1:1000, Cell Signaling Technology, Poston, USA), Runx2 (1:1000, CST), BSP II (1:1000, CST), Dlx1 (1:200, Biorbyt), Msx1 (1:200, Biorbyt), and GAPDH (1:1000, CST) were used as internal standards. On the second day, the membranes were incubated with anti-rabbit or anti-mouse secondary antibody/HRP (1:5000, Bioss) for two hours at room temperature. Finally, the signal was revealed with BeyoECL Plus solution (Beyotime) by the imaging system (Bio-Rad). In order to see the difference clearly, the Quantity One software (Bio-Rad; Hercules, USA) was used to grayscale analysis for the blots and the ratio of the grayscale value of target blot and that of internal reference GAPDH blot was considered as the relative expression level.

### Alkaline phosphatase and alizarin red staining

Each group cells were cultured with the osteogenic induction medium. On days 14 and 21, the cells were fixed with 4% paraformaldehyde and stained using an alkaline phosphatase (Alp) staining kit (Beyotime, Shanghai, China) or alizarin red (Sangon Biotech, Shanghai, China) staining kit separately, according to the manufacturer’s instructions and the previous study^1^. The stained images were captured by a stereo fluorescence microscope (Zeiss).

### Statistical analysis

Total data are shown as the mean ± standard deviation (SD). Statistical significance was determined with SPSS 20.0 software (IBM Analytics, Armonk, USA) and GraphPad Prism8 software (GraphPad software, california, USA). The comparison between groups in our study were pairwise comparison. Following the determination of normal distribution by *F*-test, normally distributed data were analyzed by unpaired *T* test and non-normally distributed data were analyzed by Mann–Whitney test. Significance levels of *p* < 0.05 indicated significant differences.

### Statement

The study is reported in accordance with ARRIVE guidelines.

## Supplementary Information

Supplementary Information.

Supplementary Table 2.

Supplementary Table 3.

Supplementary Table 4.

Supplementary Table 5.

## Data Availability

All data generated or analysed during this study are included in this published article and its supplementary information files.
