# Supplementary material for: Dynamic expression of Mage-D1 in rat dental germs and potential role in mineralization of ectomesenchymal stem cells
Source: Sci Rep. 2022 Dec 30;12:22615. doi: 10.1038/s41598-022-27197-5 (PMC9803661; doi:10.1038/s41598-022-27197-5)
Supplement: Supplementary file 1 — Supplementary Information. [file 41598_2022_27197_MOESM1_ESM.docx]

**Dynamic expression of Mage-D1 in rat dental germs and potential role in mineralization of ectomesenchymal stem cells**

**Meng Li^1^, Xia Yu^2^, Yuting Luo^1^, Hongyan Yuan^2^, Yixing Zhang^2^, Xiujie Wen*^2^, Zhi zhou*^1^**

Supplementary Table 1. Specific primers used for RT-PCR.

| **Gean** | **Primer sequences** |
| --- | --- |
| GAPDH | Forward: 5’‐AAGTTCAACGGCACAGTCAAGG‐3’ |
|  | Reverse: 5’‐ACGCCAGTAGACTCCACGACAT‐3’ |
| Runx2 | Forward: 5’‐CTGCCACCTCTGACTTCTGC‐3’ |
|  | Reverse: 5’‐GATGAAATGCCTGGGAACTG−3’ |
| Col-1 | Forward: 5’‐GGTCCTTCTGGTCCTCGTG‐3’ |
|  | Reverse: 5’‐TCTCCGTTCTTGCCAGGA‐3’ |
| ALP | Forward: 5’‐GGCTCTGCCGTTGTTTCTCT‐3’ |
|  | Reverse: 5’‐AAGGTGCTTTGGGAATCTGC‐3’ |
| p75NTR | Forward: 5’‐GAGGGCACATACTCAGACGA‐3’ |
|  | Reverse: 5′‐CTCTTCGCATTCAGCATCAG‐3’ |
| Dlx1 | Forward: 5'-CAGCCCCTACATCAGTTCCG -3' |
|  | Reverse: 5'-CTTCTCCGCCTTCCACCAC-3 |
| Msx1 | Forward: 5'-CACCCTACGCAAGCACAAGAC-3' |
|  | Reverse: 5'-CGCTCGGCAATAGACAGGTAC-3' |
| Dspp | Forward: 5’‐GAGCAGCCTCATCAGGATACAAA‐3’ |
|  | Reverse: 5’‐CTGGTTCTCACTCCCCTCCC‐3’ |
| Dmp1 | Forward: 5’‐AAAACAGTGCCCAAGATACCCC‐3’ |
|  | Reverse: 5’‐CCACCTCCTACCCGATATTCCT‐3’ |
| Mage-D1 | Forward: 5’‐ GCCGTCCTCTGGGAAGCA‐3’ |
|  | Reverse: 5’‐ GCTGTTGGGCACTCGTCTGT‐3’ |

Supplementary Figure S1.


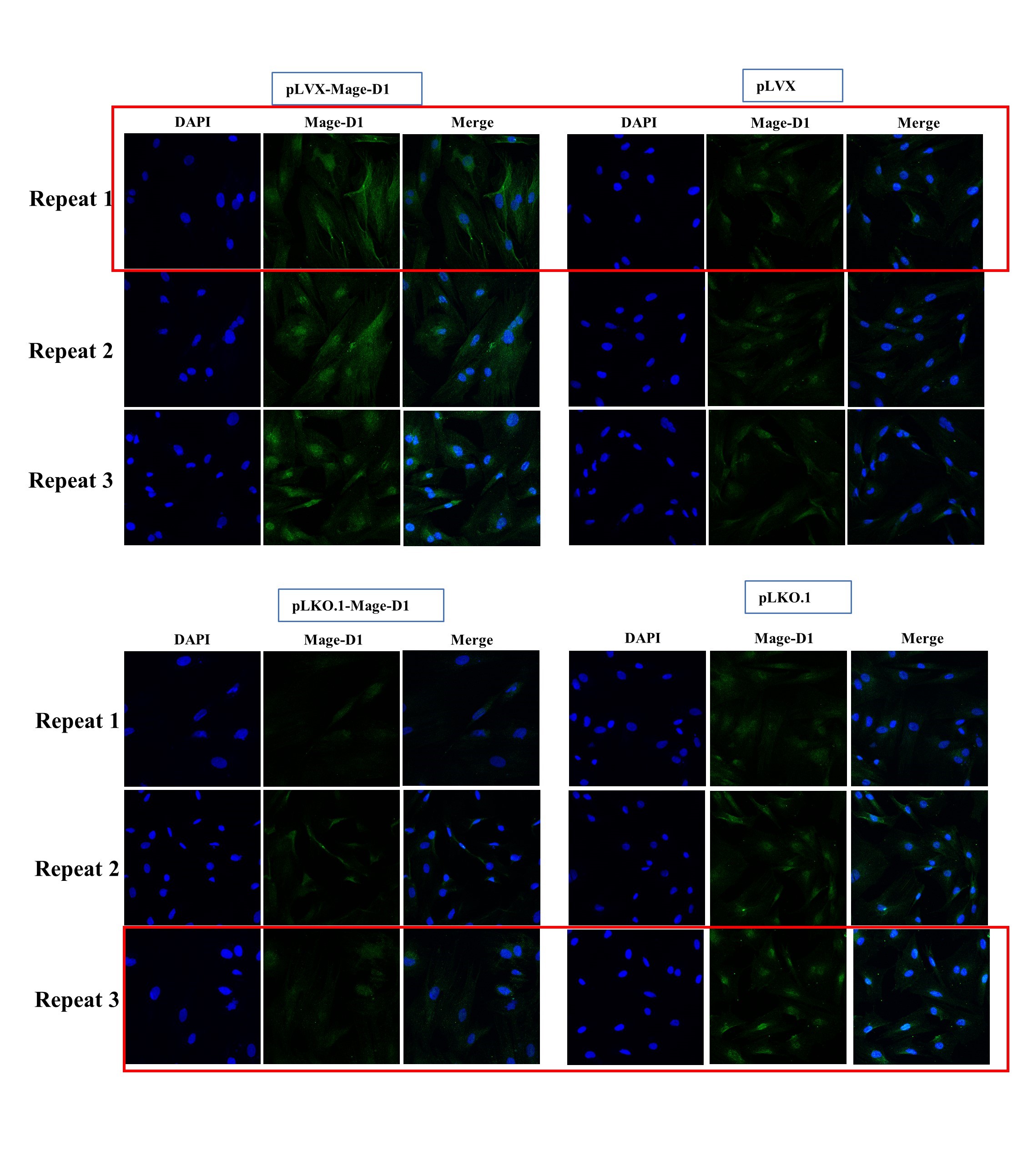


Figure S1. Immunocytofluorescence staining of empty plasmid (pLVX group), transfection with Mage-D1 overexpression plasmid pLVX (pLVX Mage-D1 group), empty plasmid (Plko.1 group) and transfection with Mage-D1 silence plasmid pLKO.1 (pLKO.1-Mage-D1 group); scale bar represents 25 μm. This experiment was repeated three times.

Supplementary Figure S2.


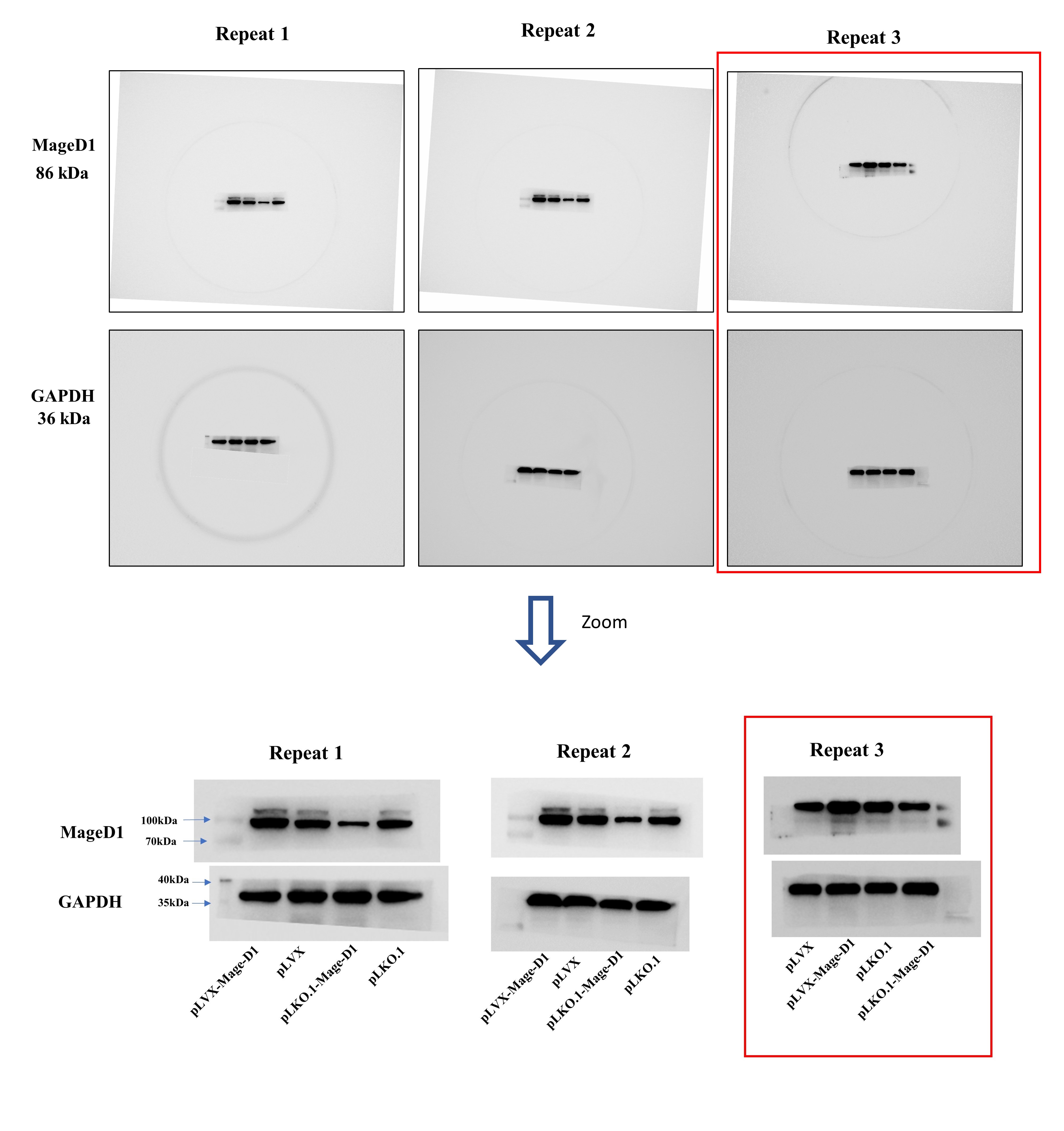


Figure S2. After transfection of cells with lentivirus, the expression levels of Mage-D1 were detected by Western blot analysis, GAPDH used as the reference gene. Empty plasmid (pLVX group), transfection with Mage-D1 overexpression plasmid pLVX (pLVX Mage-D1 group), empty plasmid (Plko.1 group) and transfection with Mage-D1 silence plasmid pLKO.1 (pLKO.1-Mage-D1 group). This experiment was repeated three times.

Supplementary Figure S3.


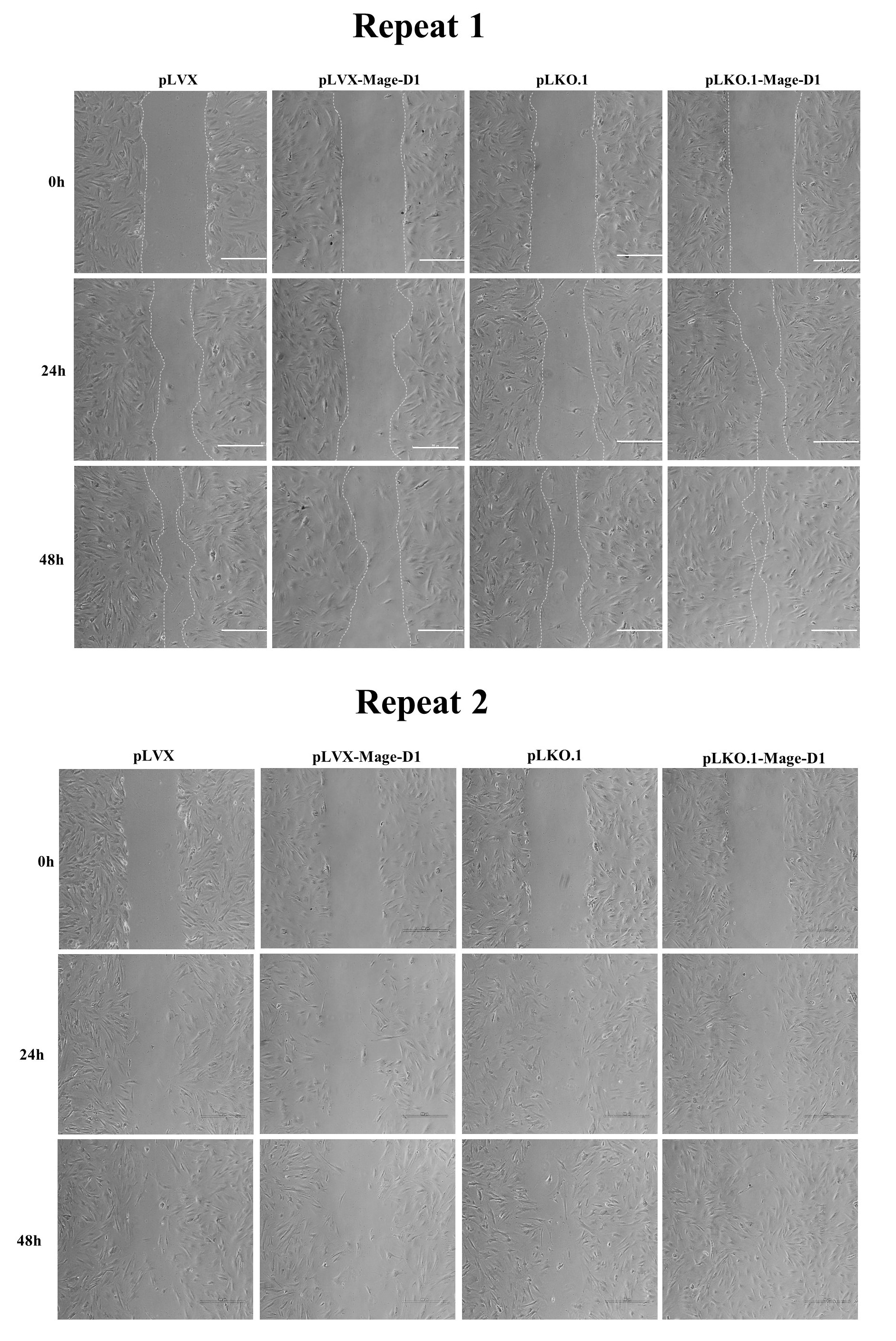


Figure S3. The migration rate was assessed for two consecutive days. Scale bar represents 500 μm. This experiment was repeated two times.

Supplementary Figure S4.


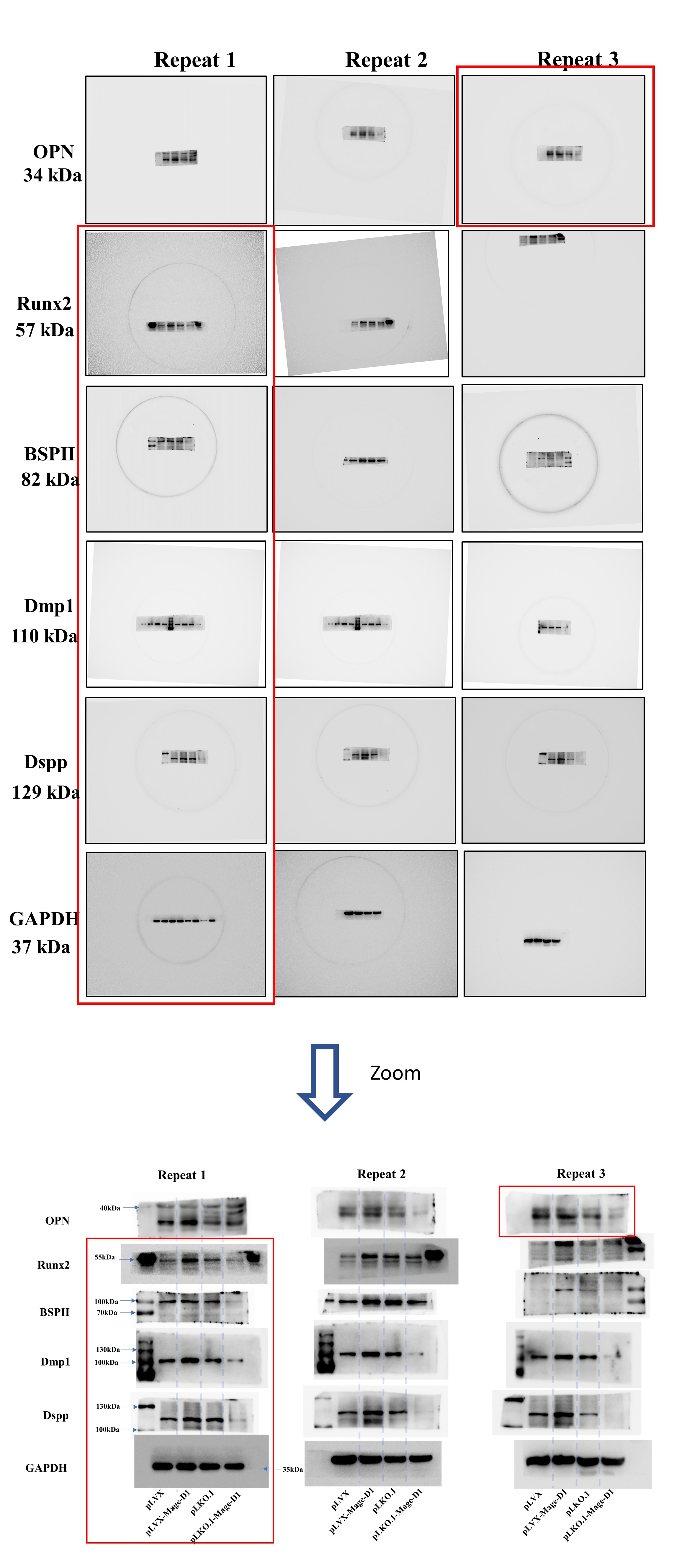


Figure S4. Under induction with mineralized culture medium for 14 d, the expression levels of OPN, Runx2, BSPⅡ, Dmp1, Dspp were detected by Western blot analysis, GAPDH used as the reference gene. This experiment was repeated three times.

Supplementary Figure S5.


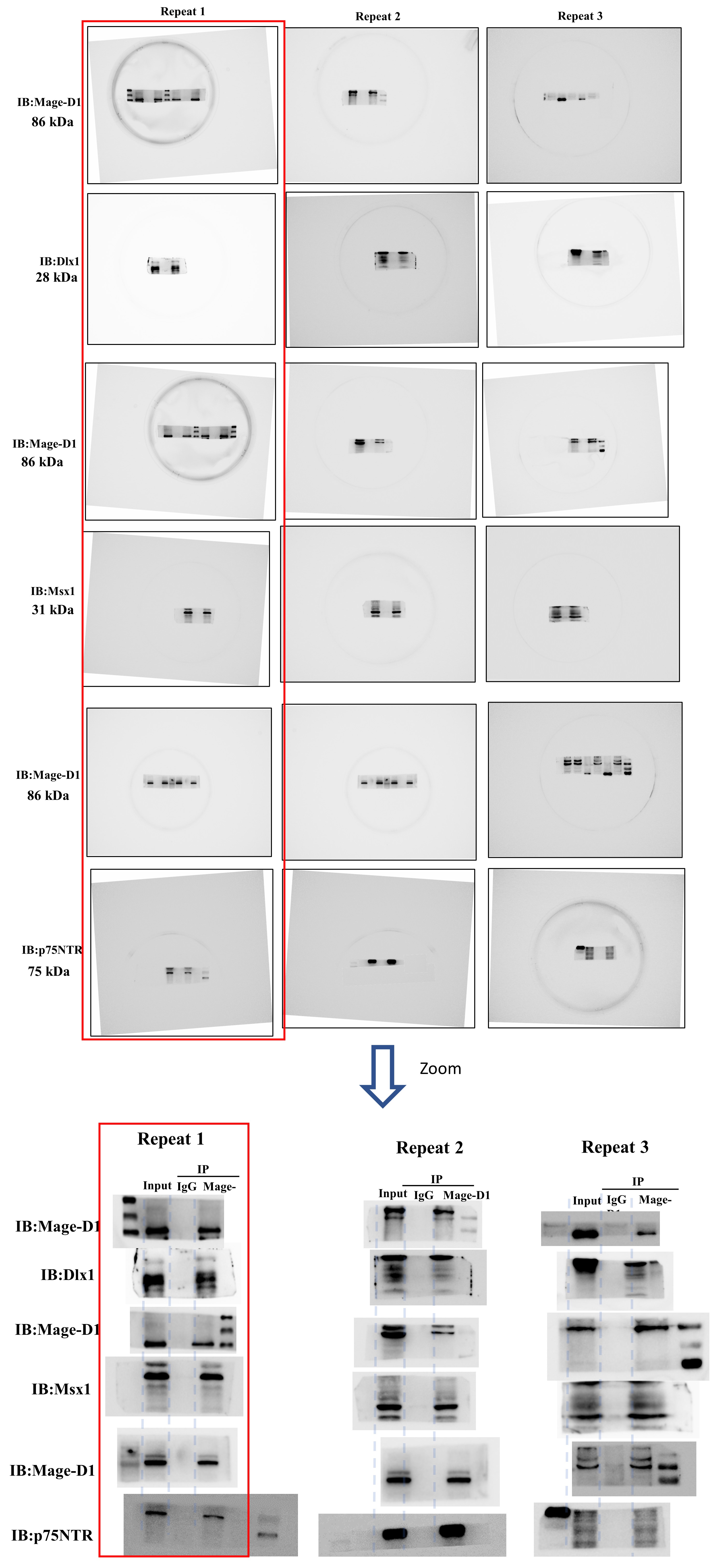


Figure S5. Under induction with mineralized culture medium for 14 d, the expression levels of p75NTR, Msx1, Dlx1 were detected by Western blot analysis, GAPDH used as the reference gene. This experiment was repeated three times.

Supplementary Figure S6.


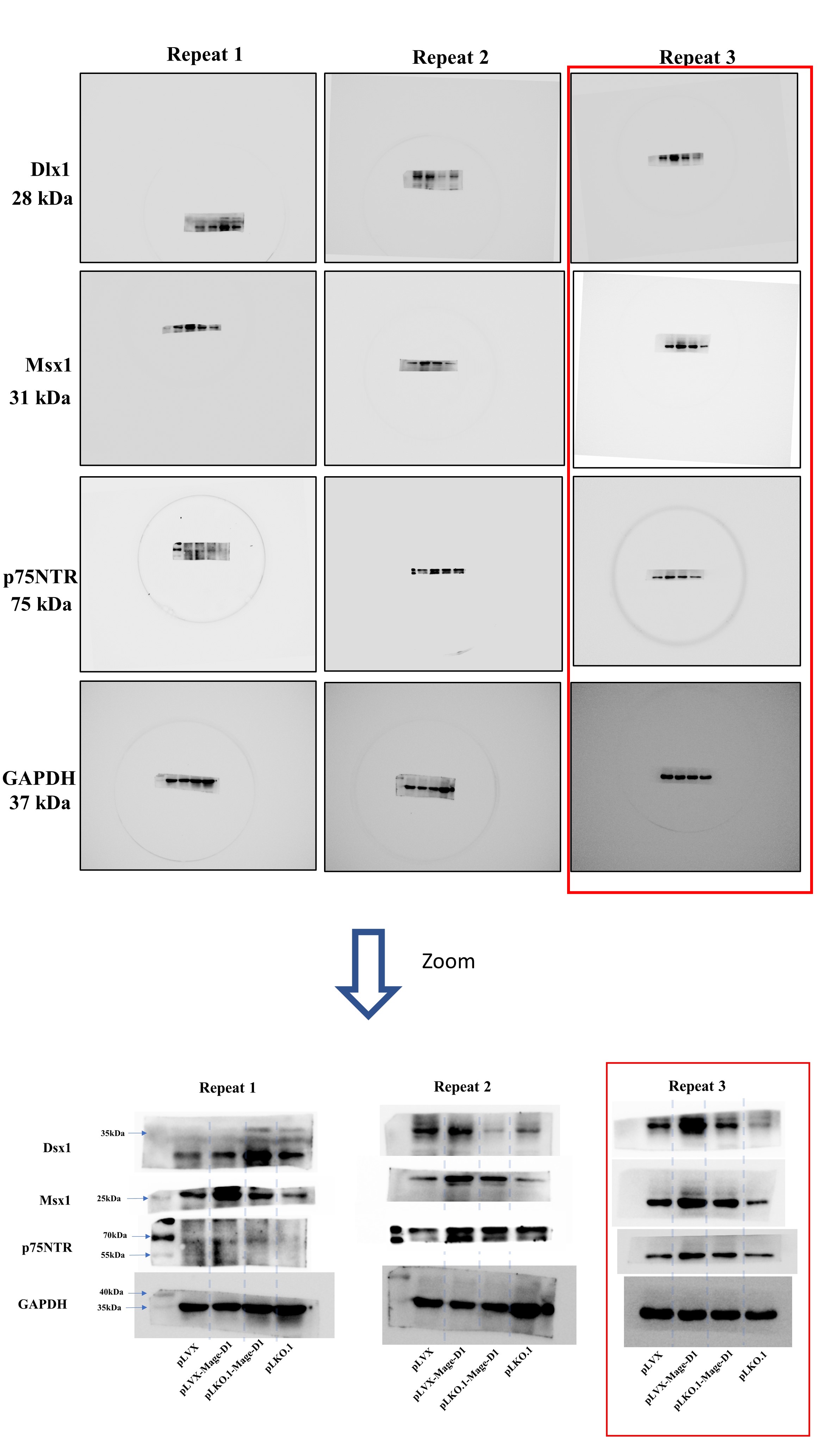


Figure S6. Co-immunoprecipitation shows that Mage-D1 can bind to p75NTR, Msx1, Dlx1 based on E19.5 d EMSCs. This experiment was repeated three times.
